# Criteria for Drugs Used in Pre-Exposure Prophylaxis Trials against HIV Infection

**DOI:** 10.1371/journal.pmed.0030454

**Published:** 2006-11-07

**Authors:** Inge Derdelinckx, Mark A Wainberg, Joep M. A Lange, Andrew Hill, Yasmin Halima, Charles A. B Boucher

## Abstract

The authors formulate criteria for an optimal pre-exposure prophylaxis drug candidate, and evaluate existing antiviral drug classes for their suitability.

In 2004, almost 5 million people became newly infected with HIV, emphasizing the continuous need for effective prevention strategies. The development of an effective preventive vaccine faces many difficulties and is not likely to occur within the next decade. Behavioural changes, such as consistent and correct condom use and abstinence from high-risk behaviour, probably contributed to the fall in HIV incidence recently reported in several African countries and in India [1]. Recently, it was shown that male circumcision reduced the risk of acquiring HIV by 60% [2] and the risk of male-to-female transmission by 30% [3]. However, behavioural interventions may not be able to curb the HIV epidemic as much as needed, prompting the need to find additional, more effective preventive strategies. There is also a need for “female-initiated intervention”. Women around the globe continue to be infected with HIV by their male partners and often feel unable to insist on condom use.

Alternatives to behavioural strategies include those that are based on drugs. Anti-HIV vaginal microbicides, which may offer women a means of protecting themselves from infection, are currently being evaluated [4]. Also, rectally applied microbicides have proven to effectively prevent infection in macaques challenged via this transmission route [5]. However, like male circumcision, this strategy may not provide protection against other routes of HIV transmission, such as oral or intravenous transmission.

Oral antiretroviral pre-exposure prophylaxis (PREP) in high-risk populations may be a more reliable tool in preventing transmission of HIV [6]. Limited animal model data suggest that antiretroviral drugs may prevent infection when taken prior to, at the time of, and/or after HIV exposure [7–10]. Theoretically, preventing HIV infection could be done by blocking any step in the HIV life cycle. Blocking a step prior to integration of proviral DNA into the host DNA is believed to have greater potential since this way the permanent integration of proviral HIV is averted.

## The Initial PREP Trials

For the initial trials studying the safety and efficacy of PREP, tenofovir disoproxil fumarate (TDF) was selected, based on its encouraging data from animal studies. So far, no other antiretroviral drug has been evaluated in human PREP studies. However, there is no reason why PREP research should be restricted to TDF.

In the absence of animal data, other drugs with antiviral effects similar to TDF represent possible alternatives. Also, recent data seem to show rapid in vitro selection of K65R mutation in HIV subtype C (the most prevalent subtype worldwide) compared to other subtypes, suggesting the need for caution when using the drug on a wide scale [11]. Recent results also suggest that the protective effect of TDF monotherapy may be overcome by repeated exposures to the virus in animal models [12], providing further impetus not to focus on TDF alone in PREP research.

In this paper, we formulate criteria for an optimal PREP drug candidate based on a literature review and our expert opinion where appropriate. Also, we evaluate existing antiviral drug classes for their suitability in PREP.

## Criteria for PREP Drug Candidates

The biological basis for PREP is that the drug/regimen is capable of averting new HIV infections. In the absence of data from randomised controlled clinical trials, this requirement, as well as some others, can be translated into specific criteria that should be met by a PREP drug.

**Box 1. PREP Antiviral Criteria**

**Assured Effectiveness**

- *High genetic barrier*: PREP drugs should require multiple mutations to cause virologic failure (principle of high genetic barrier). In this case, the likelihood of pre-existing resistant viral strains in the inoculum is low.
- *Unique resistance profile*: Ideally, there is no or limited cross-resistance between the resistance profile associated with the PREP regimen and those associated with other antiviral drugs, guaranteeing the effectiveness of the PREP regimen in settings with some levels of resistance at population level. Since all currently available antivirals have some cross-resistance with other drugs, this criteria is not being met by any of the current antivirals, but the cross-resistance should be kept minimal.

**Limiting the Impact of Selected Drug Resistance on Future Treatment Response**

In the situation that the individual becomes inadvertently infected during PREP, there is a risk of selection of drug-resistant viruses. It is unclear if/how this may affect future treatment response, but this impact should at any rate be minimised and be weighed against the benefits of PREP. On a theoretical basis, the selected drug-resistant viruses should therefore meet the following criteria:

- *Reduced transmissibility*: If the selected resistant viral strains are less transmissible compared to wild-type viruses, this may reduce the secondary spread of the resistant virus to other individuals. Also, this may permit the drug to retain antiviral activity as a PREP agent even in populations in which many potential HIV transmitters are infected with viruses resistant to the drug.
- *Reduced replication capacity*: If the selected resistant viral strains have a reduced replication fitness compared to wild type, this will result in the preferential replication of the drug-sensitive virus after discontinuation of the drug in the infected individual. Over time, the drug-resistant virus could be reduced to very low levels, comparable with those present in the natural variation of a wild-type quasi-species. These low levels of drug-resistant variants may affect treatment response favorably when compared with resistance present as a major variant. Reduced replication capacity is generally related to reduced viral load levels, which is associated with reduced transmissibility [18,32].
- *Unique resistance profile*: If the selected resistant viral strains have a unique resistance pattern not shared by other antiretroviral drugs, this will reduce the impact on possible future treatment response.
- *High effectiveness of PREP regimen*: The effectiveness of the PREP regimen at preventing a new HIV infection should be high. If PREP reduces the number of new infections in a population, the number of potential resistant strains developed will be low.

**Safety profile.** Safety is an important issue for all drugs used in clinical practice, especially in individuals without disease. Since a PREP drug may be taken regularly over a very long time period (years), the best candidate drugs should have a favorable safety profile based on extensive clinical experience and large safety databases.

**Ease of use.** PREP is only likely to be successful as a strategy if it can be easily adopted into daily life. No formal criteria for “ease of use” have been defined for the PREP setting, but ease of use would require convenient drug dosing options, such as once daily or once weekly dosing, a limited number of pills, the absence of strict food regulations when taking the drug, and excellent tolerability. Ideally, PREP should offer continued protection even if an occasional dose is missed. Thus, the pharmacokinetic and toxicity profiles of the compound will be critical to the success of the PREP strategy.

**Mode of action and pharmacology.** Theoretically, prevention of integration of the proviral DNA into the host cell genome during the very first replicative cycle is the preferred mode of action. If the viral genome has become integrated in the host cell, there is a danger that infectious progeny could be expressed at a later stage and the individual would become infected. However, recent data suggest that a minimal seeding population of infected cells is required in the mucosa for infection to become established [13]. Therefore, reducing this seeding population or its dissemination of progeny virus may be enough to block establishment of infection. From a pharmacological perspective, the active metabolite of the candidate drug should achieve sufficiently high drug levels at the sites of virus entry into the body and at sites of viral replication and should be active in all cell types that are relevant to HIV transmission. However, adequate levels for the prevention of HIV transmission are not known at present.

**Antiviral profile.** First, the PREP compound should retain its preventive effect against drug-resistant viral strains selected by other antiviral drugs used in the same geographic area. Second, in case the PREP regimen fails to prevent infection with HIV, and drug-resistant viral strains are selected in this process, the impact of this drug resistance on subsequent treatment response should be minimised. The criteria that should apply to a potential PREP drug regarding its antiviral profile are shown in Box 1.

**Cost-effectiveness.** In order for PREP treatment to be widely introduced, the cost-effectiveness ratio needs to compare favorably with other common medical interventions or procedures in developing countries. The cost-effectiveness of PREP depends on three key factors: (1) the price of the PREP treatment used (US$ per person-year of treatment); (2) the efficacy of the PREP (percentage reduction in incidence of HIV infections for PREP treatment versus placebo); and (3) the incidence of new HIV infections per year in populations targeted for PREP treatment.

In order to be cost-effective, the cost of PREP per infection avoided should be lower than the estimated lifetime treatment cost of an HIV-infected individual.

## Evaluation of PREP Drug Candidates Based on the Formulated Criteria

Table 1 shows how the various PREP candidates compare based on the above criteria. TDF was the first antiviral drug studied in PREP clinical trials, based upon pre-clinical data showing significant protection against simian immunodeficiency virus and simian HIV infection when TDF was dosed prior to or after viral exposure in small transmission intervention studies in simian models [7,8]. The oral formulation of TDF meets most of the criteria formulated above for appropriate PREP candidates. In this article we evaluate other antivirals for their potential as good PREP drug candidates and we make the case that lamivudine (3TC) may be a useful PREP drug. Given the similarity in structure and antiviral profile between 3TC and emtricitabine (FTC), all the arguments made for 3TC are also valid for FTC, although experience with FTC is more limited.

**Table 1:**
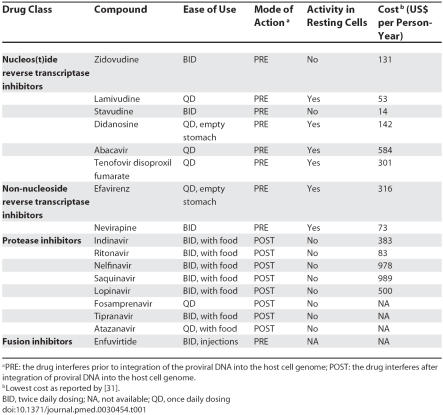
Summary of Some PREP Criteria for Antiviral Compounds Approved for Treatment of HIV Infection

**Safety profile.** Antivirals are known to have various side effects. Efavirenz often causes central nervous system side effects. Nevirapine can have life threatening side effects (liver toxicity, Stevens-Johnson syndrome). The use of protease inhibitors often leads to lipodystrophy and other metabolic disorders. Nucleoside reverse transcriptase inhibitors (NRTIs) come with specific side effects depending on the drug. 3TC is by far the best tolerated and least toxic HIV drug currently approved for patient treatment, with the best safety record over the longest period of time. In addition, 3TC has been widely used in prevention of mother-to-child transmission studies and has also been taken by many women who became pregnant while on therapy, without apparent injury to newborns or the fetus.

**Ease of use.** Most antivirals can be administered twice daily, and many can be administered once a day. However, only three drugs can be administered once daily without food restrictions: TDF, 3TC, and FTC.

**Mode of action and pharmacology.** Non-nucleoside reverse transcriptase inhibitors (NNRTIs), NRTIs, and fusion inhibitors all halt viral replication prior to integration of viral DNA into host cell DNA. Since protease inhibitors halt replication after integration takes place, they are theoretically less favourable PREP candidates. 3TC acts early in the HIV replication cycle, before integration, and appears to retain antiviral potency in both activated and non-activated cells. Phosphorylation of 3TC to the active triphosphorylated compound does not seem to be restricted by either the type of host cell or the activation stage of the cells [14].

**Antiviral profile.** A successful PREP candidate will need to have a unique resistance profile. Resistance selected by protease inhibitors and NNRTIs generally gives rise to broad cross-resistance to all other drugs in the class. In addition, thymidine-associated mutations selected by NRTIs confer cross-resistance to various other NRTIs. 3TC readily selects for the M184I/V mutations. These mutations confer selective resistance, which is largely restricted to 3TC/FTC. Although low-level cross-resistance can be conferred by the M184V mutation in vitro against didanosine (DDI) and abacavir, clinical studies suggest that the selection of the this mutation does not impact the subsequent virologic response to DDI [15,16]. For most other nucleos(t)ides (zidovudine, stavudine, TDF) the antiviral activity of the drugs in the presence of M184V is slightly enhanced [17]. In contrast, the resistance profile associated with TDF therapy includes mutations, such as K65R and T215Y, that are associated with broader cross-resistance across the nucleoside class of drugs.

The candidate should reduce HIV transmissibility. Various factors are known to be associated with an increased transmission of HIV: high viraemia [18], concomitant sexually transmitted diseases [19], host genetic factors [20], and behavioural characteristics. However, little is known about the factors associated with the selective transmission of drug-resistant viruses. A recent study comparing the prevalence of drug resistance mutations among viruses from primary HIV-1 infection (PHI) with viruses from a representative population of potential transmitters found that the prevalence of M184V and major protease inhibitor mutations was significantly lower in the PHI group. For M184V this difference was most striking: a prevalence of 10.1% in the PHI group versus 70.0% in the treated group [21]. This was confirmed in a European dataset [22]. These data suggest a diminished transmission of these HIV-1 variants, which may reflect both their diminished replicative and/ or transmission capacity as well as their potential consequence on the viraemia. Early reversion to wild-type virus cannot be excluded; however, the PHI group included patients infected during the last six months based on strict criteria and long-term persistence of transmitted resistance in PHI has been shown [23,24].

The candidate must also demonstrate proof of principle and effectiveness. In the SIMBA study, conducted in Rwanda and Uganda, 3TC was administered on a daily basis to HIV-negative infants from HIV-positive breast-feeding mothers (who had received pre-partum zidovudine/DDI) for the duration of breast-feeding (maximum six months). In contrast to earlier results in the Petra study, with a similar pre-partum maternal intervention, but with no infant PREP during breast-feeding, virtually no infant infections occurred in the SIMBA study [25]. Similar results have recently been presented from a study conducted in Tanzania [26].

The candidate should reduce the replication capacity of the virus. A number of reverse transcriptase and protease mutations affect the replication capacity of HIV. The NRTI resistance mutation M184V and the protease inhibitor mutation D30N are single mutations associated with reduced viral replication compared to wild-type virus. These mutant variants usually become undetectable within weeks of discontinuing therapy and are frequently replaced by wild-type virus, indicating their in vivo reduced fitness in the absence of drugs compared to the wild-type virus. In the case of the protease inhibitor–resistant virus, the initial reduction of replication capacity can be compensated by the selection of additional mutations [27]. The reduced replication capacity of the virus granted by the M184V mutation persists and is often associated with a persistent partial antiviral effect during continued administration of the drug [28]. The diminished replication fitness of M184V-containing viruses is thought to be related to the location of the M184V amino acid residue close to the active site of the viral reverse transcriptase enzyme. The mutation is thought to interfere with the replication process of the reverse transcriptase enzyme through a diminished transcription processivity, an increased transcription fidelity resulting in a decreased mutation rate, a diminished initiation of the reverse transcription reaction, and a diminished rate of excision of incorporated nucleotides from viral DNA [29].

## Cost-Effectiveness

Hill and co-authors did an analysis to determine the conditions under which the cost of PREP treatment would be lower than the cost of treating people for life with highly active antiretroviral therapy once HIV infection occurred in a sub-Saharan African setting without access to PREP [30]. They estimated that the lifetime treatment cost was US$10,000 over 20 years ($500 estimated per year, to cover both diagnostics and medical expenses).

The cost per HIV infection prevented by PREP was calculated for various HIV incidences (between 1%–15%) and for different costs of the PREP drug ($50, $100, $250, and $500). The results are depicted for two efficacy rates: see Figure 1, where PREP is assumed to cut incidence of HIV by 40%, and Figure 2, where this efficacy rate is set at 80%.

The lowest annual drug costs included $53 per person-year for generic 3TC, $200 for the standard generic treatment regimen stavudine/3TC/nevirapine, $300 for TDF, and $500 for lopinavir/ ritonavir [31]. With an HIV incidence of 1%, and assuming 80% efficacy of PREP, 125 people would require PREP treatment to prevent one HIV infection, resulting in a cost per infection avoided, in the case of generic 3TC being used, of $6,625. In these settings, PREP with generic 3TC would be cost-effective (costing less than $10,000).

**Figure 1:**
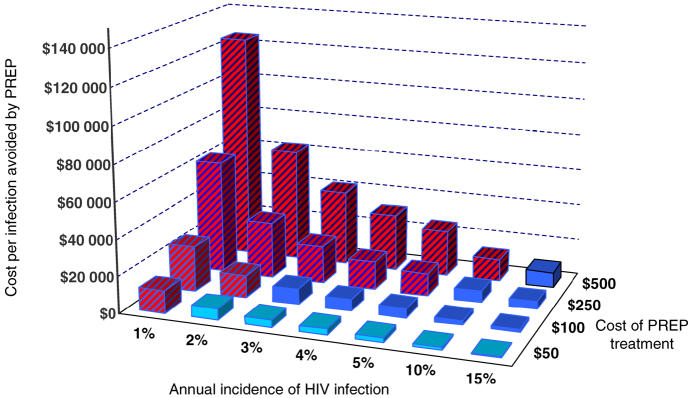
Cost-Effectiveness of PREP with a 40% Efficacy Rate This graph shows the cost per infection avoided by PREP (US$) by annual HIV incidence (%) and by cost of PREP treatment (US$), assuming PREP cuts incidence of HIV infection by 40%. The striped bars indicate that the cost per infection avoided by PREP was over $10,000 and was therefore not considered to be cost-effective.

**Figure 2:**
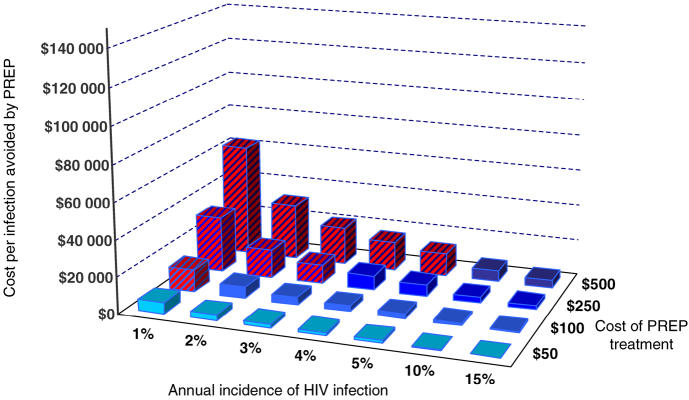
Cost-Effectiveness of PREP with an 80% Efficacy Rate This graph shows the cost per infection avoided by PREP (US$) by annual HIV incidence (%) and by cost of PREP treatment (US$), assuming PREP cuts incidence of HIV infection by 80%. The striped bars indicate that the cost per infection avoided by PREP was over $10,000 and was therefore not considered to be cost-effective.

In addition, 3TC is also included in the World Health Organization list of essential drugs for HIV treatment in the developing world. Thus, the relevant developing country stakeholders for PREP clinical trials and PREP approval are already familiar with 3TC and have experience in its use.

## Combined PREP (“Combo-PREP”)

Recently, promising results were shown when combining TDF with FTC as PREP, the so-called combo-PREP, in an animal model [10]. None of six male rhesus macaques who were receiving both drugs became infected when repeatedly rectally exposed to simian HIV, compared to five of six male control macaques who were receiving no treatment. These results prompted some of the ongoing and planned PREP trials to be modified to test Truvada, the combination tablet of TFD and FTC, instead of a single PREP drug. Results of these trials will help elucidate the value of using more than one drug in PREP regimens. These results should be interpreted by also looking at possible downsides of combinations of drugs, such as increased toxicity and cost.

## Issues Related to Designing PREP Studies

A randomised trial studying the incidence of new HIV infections in a group treated with PREP compared to placebo will address the efficacy and safety of the drug to prevent HIV infection. Although PREP would be most beneficial and cost-effective in the developing world where there is high HIV prevalence, a proof of concept study is urgently needed and should be organized irrespective of the setting. Large numbers should be included, especially in low HIV incidence settings, given that the continued counseling on use of other proven prevention measures will most certainly also reduce HIV incidence.

Several ethical and logistical issues need to be carefully addressed when designing a PREP trial, especially in resource-poor settings. What about the treatment and care of participants who become infected during the program and of those excluded from the trial because they were found to be HIV positive at baseline? Proven effective prevention interventions should be delivered to participants, and mechanisms to promote research literacy for host communities put in place. The studies should be carefully discussed and planned in collaboration with local communities. In addition, in case PREP proves to be efficacious and would be implemented on a large scale, social marketing will be very important to promote this prevention measure along with others and to educate people on proper use of PREP and expected outcomes.

## 3TC as PREP: Issues for Consideration

Based on the above stated criteria, 3TC would be an interesting PREP drug candidate. However, two areas need attention when using 3TC as a PREP drug. First, the impact on future treatment response of individuals infected when exposed to 3TC monotherapy should be carefully monitored since the currently available drug regimens in resource-poor settings generally include 3TC. It remains unclear whether archived 3TC resistance, overgrown by wild-type virus due to its diminished replicative capacity, would be again selected by 3TC as part of the antiretroviral treatment regimen and thus have an impact on subsequent treatment response.

Secondly, the impact of 3TC on the course of chronic hepatitis B infection should be closely monitored. 3TC was the first nucleoside analogue licensed for treatment of chronic hepatitis B infection. However, after an initial response, resistant hepatitis B virus could be selected, resulting in a breakthrough infection which may be accompanied by acute exacerbation of liver disease. Also, acute exacerbation of hepatitis with or without hepatic decompensation may occur after discontinuation of 3TC treatment, as is the case with TDF. On the other hand, these drugs may be effective in prevention of hepatitis B virus infection as well as that of HIV.

## Conclusion

In this paper, we have formulated criteria to which a PREP drug should adhere based on theoretical grounds and expert opinion. According to these criteria, we have argued that 3TC would make an interesting PREP drug given its relative safety, ease of use, mode of action and pharmacology, antiviral profile, and cost-effectiveness. Whether its use will be optimal as monotherapy, or in combination with another antiviral, is still unknown. Randomised clinical trials to evaluate 3TC's efficacy in preventing new HIV infections are the only way forward.

## Abbreviations

3TC: lamivudine
DDI: didanosine
FTC: emtricitabine
NRTI: nucleoside reverse transcriptase inhibitor
NNRTI: non-nucleoside reverse transcriptase inhibitor
PHI: primary HIV-1 infection
PREP: pre-exposure prophylaxis
TDF: tenofovir disoproxil fumarate

## References

[pmed-0030454-b001] Shelton JD, Halperin DT, Wilson D (2006). Has global incidence peaked?. Lancet.

[pmed-0030454-b002] Auvert B, Taljaard D, Lagarde E, Sobngwi-Tambekou J, Sitta R (2005). Randomized, controlled intervention trial of male circumcision for reduction of HIV infection risk: The ANRS 1265 trial. PLoS Med.

[pmed-0030454-b003] Gray R, Wawer MJ, Thoma M, Serwadda D, Nalugoda F (2006). Male circumcision and the risks of female HIV and sexually transmitted infection acquisition in Rakai, Uganda. http://www.retroconference.org/2006/Abstracts/25977.HTM.

[pmed-0030454-b004] Dhawan D, Mayer KH (2006). Microbicides to prevent HIV transmission: Overcoming obstacles to chemical barrier protection. J Infect Dis.

[pmed-0030454-b005] Tsai C-C, Emau P, Jiang Y, Tian B, Morton WR (2003). Cyanovirin-N gel as a topical microbicide prevents rectal transmission of SHIV89.6P in macaques. AIDS Res Hum Retroviruses.

[pmed-0030454-b006] Youle M, Wainberg MA (2003). Could chemoprophylaxis be used as an HIV prevention strategy while we wait for an effective vaccine?. AIDS.

[pmed-0030454-b007] Tsai C-C, Follis KE, Sabo A, Beck T, Grant RF (1995). Prevention of SIV Infection in Macaques by (R)-9(2-Phosphonylmethoxyprop yl)adenine. Science.

[pmed-0030454-b008] Van Rompay KK, McChesney MB, Aguirre NL, Schmidt KA, Bischofberger N (2001). Two low doses of tenofovir protect newborn macaques against oral simian immunodeficiency virus infection. J Infect Dis.

[pmed-0030454-b009] Veazey RS, Springer MS, Marx PA, Dufour J, Klasse PJ (2005). Protection of macaques from vaginal SHIV challenge by an orally delivered CCR5 inhibitor. Nat Med.

[pmed-0030454-b010] Garcia-Lerma JG, Otten R, Qari SH, Jackson E, Luo W (2006). Prevention of rectal SHIV transmission in macaques by tenofovir/FTC combination. http://www.retroconference.org/2006/Abstracts/27976.

[pmed-0030454-b011] Brenner BG, Oliveira M, Doualla-Bell F, Moisi D, Ntemgwa F (2006). HIV-1 subtype C viruses rapidly develop K65R resistance to tenofovir in cell culture. AIDS.

[pmed-0030454-b012] Subbarao S, Otten R, Ramos A, Jackson E, Adams D (2005). Chemoprophylaxis with oral tenofovir disoproxil fumarate (TDF) delays but does not prevent infection in rhesus macaques given repeated rectal challenges of SHIV. http://www.aegis.com/conferences/croi/2005/136LB.html.

[pmed-0030454-b013] Miller CJ, Li Q, Abel K, Kim EY, Ma ZM (2005). Propagation and dissemination of infection after vaginal transmission of Simian Immunodeficiency Virus. J Virol.

[pmed-0030454-b014] van 't Wout AB, Ran LJ, Nijhuis M, Tijnagel JM, Groot TD (1998). Efficient inhibition of both syncytium-inducing and non-syncytium-inducing wild-type HIV-1 by lamivudine in vivo. AIDS.

[pmed-0030454-b015] Marcelin A-G, Fandre P, Pavie J, Schmidely N, Wirden M (2005). Clinically relevant genotype interpretation of resistance to didanosine. Antimicrob Agents Chemother.

[pmed-0030454-b016] Sproat M, Pozniak AL, Peeters M, Winters B, Hoetelmans R (2005). The influence of the M184V mutation in HIV-1 reverse transcriptase on the virological outcome of highly active antiretroviral therapy regimens with or without didanosine. Antivir Ther.

[pmed-0030454-b017] Whitcomb JM, Parkin NT, Chappey N, Hellmann N, Petropoulos CJ (2003). Broad nucleoside reverse-transcriptase inhibitor cross-resistance in human immunodeficiency virus type 1 clinical isolates. J Infect Dis.

[pmed-0030454-b018] Quinn TC, Wawer MJ, Sewankambo N, Serwadda D, Li C (2000). Viral load and heterosexual transmission of human immunodeficiency virus type 1. Rakai Project Study Group. N Engl J Med.

[pmed-0030454-b019] Cohen MS (1998). Sexually transmitted diseases enhance HIV transmission: No longer a hypothesis. Lancet.

[pmed-0030454-b020] Katzenstein TL, Eugen-Olsen J, Hofmann B, Benfield T, Pedersen C (1997). HIV-infected individuals with the CCR delta32/CCR5 genotype have lower HIV RNA levels and higher CD4 cell counts in the early years of the infection than do patients with the wild type. Copenhagen AIDS Cohort Study Group. J Acquir Immune Defic Syndr Hum Retrovirol.

[pmed-0030454-b021] Turner D, Brenner B, Routy JP, Moisi D, Rosberger Z (2004). Diminished representation of HIV-1 variants containing select drug resistance-conferring mutations in primary HIV-1 infection. J Acquir Immune Defic Syndr.

[pmed-0030454-b022] van de Vijver DAMC, Wensing AMJ, Asjo B, Bruckova M, Jorgensen Bruun (2005). Selective transmission of drug resistant mutations. Antivir Ther.

[pmed-0030454-b023] Brenner BG, Routy JP, Petrella M, Moisi D, Oliveira M (2002). Persistence and fitness of multidrug-resistant human immunodeficiency virus type 1 acquired in primary infection. J Virol.

[pmed-0030454-b024] Delaugerre C, Morand L, Chaix ML, Picard O, Marcelin A-G (2004). Persistence of multidrug-resistant HIV-1 without antiretroviral treatment 2 years after sexual transmission. Antivir Ther.

[pmed-0030454-b025] Vyankandondera J, Luchters S, Hassink E, Pakker N, Mmiro F (2003). Reducing risk of HIV-1 transmission from mother to infant through breastfeeding using antiretroviral prophylaxis in infants (SIMBA-study). http://www.aegis.org/conferences/IASHIVPT/2003/LB7.html..

[pmed-0030454-b026] Kilewo C, Karlsson K, Massawe A, Lyamya E, Lipoyoga R (2005). Prevention of mother to child transmission of HIV-1 through breastfeeding by treating infants prophylactically with lamivudine in Dar es Salaam, Tanzania. http://www.aegis.com/conferences/iashivpt/2005/TuPe5-3P01.html.

[pmed-0030454-b027] Nijhuis M, Schuurman R, de Jong D, Erickson J, Gustchina E (1999). Increased fitness of drug resistant HIV-1 protease as a result of acquisition of compensatory mutations during suboptimal therapy. AIDS.

[pmed-0030454-b028] Kuritzkes DR, Quinn JB, Benoit SL, Shugarts DL, Griffin A (1996). Drug resistance and virologic response in NUCA 3001, a randomized trial of lamivudine (3TC) versus zidovudine (ZDV) versus ZDV plus 3TC in previously untreated patients. AIDS.

[pmed-0030454-b029] Petrella M, Wainberg MA (2002). Might the M184V substitution in HIV-1 RT confer clinical benefit?. AIDS Rev.

[pmed-0030454-b030] Hill A, Youle M, Boucher CAB (2006). Cost-effectiveness analysis of ART for pre-exposure prophylaxis. http://www.retroconference.org/2006/Abstracts/25932.HTM.

[pmed-0030454-b031] Médecins Sans Frontières (2005). Untangling the web of price reductions: A pricing guide for the purchase of ARVs for developing countries. http://www.doctorswithoutborders.org/publications/reports/2005/untanglingthewebv8.pdf.

[pmed-0030454-b032] Quinones-Mateu ME, Arts EJ, Kuiken C, Foley B, Hahn B, Marx P, McCutchan F, Mellors J, Wolinsky S, Korber B (2001). HIV-1 fitness: Implications for drug resistance, disease progression, and global epidemic evolution.

